# Trispecific eFab-eIg T-cell engagers targeting HER2 and HER3

**DOI:** 10.3389/fimmu.2025.1642454

**Published:** 2025-08-27

**Authors:** Ann-Kathrin Löffler, Annika Huber, Monilola A. Olayioye, Roland E. Kontermann, Oliver Seifert

**Affiliations:** ^1^ Institute of Cell Biology and Immunology, University of Stuttgart, Stuttgart, Germany; ^2^ Stuttgart Research Center Systems Biology (SRCSB), University of Stuttgart, Stuttgart, Germany

**Keywords:** trispecific antibody, T-cell retargeting, antibody engineering, HER2, HER3, CD3, hetEHD2

## Abstract

Trispecific antibodies have emerged as molecules for enhanced cancer immunotherapy by addressing the complexity of cancer cell biology and anti-cancer immune responses. Here, we present a novel approach to generate trispecific antibodies based on the previously developed eIg technology. These trispecific antibodies comprise one Fab and two eFab moieties, fused to obtain an asymmetric eFab-eIg molecule. The design principle employs two different eFab building blocks, characterized by divergent arrangements of heterodimerizing hetEHD2 domains. Specifically, the first (inner) eFab arm comprises the hetEHD2–1 domain in the heavy chain and the corresponding hetEHD2–2 domain in one of the light chains, while in the second eFab (outer) this arrangement is reversed. The feasibility of this approach was demonstrated for a trispecific eFab-eIg T-cell engager (TCE) targeting HER2, HER3, and CD3. Importantly, the trispecific TCE retained binding activity for all three antigens and was capable of recruiting T-cells to HER2 and/or HER3-expressing cancer cells and mediating effective cancer cell killing, as shown in 2D and 3D model systems. Due to the modular architecture, this approach should be suitable to generate trispecific antibodies of any specificity and for a multitude of applications.

## Introduction

Bispecific antibodies have found increasing applications in cancer therapy (1). The majority of the approved bispecific antibodies is designed as T-cell engagers (TCEs) that simultaneously bind to a tumor-associated antigen (TAA) on the cancer cells and to the CD3 chain of the T-cell receptor (TCR) complex on T-cells. Many of these TCEs utilize a 1 + 1 stoichiometry for the TAA and CD3 chain. However, recently TCEs with a 2 + 1 stoichiometry containing two identical binding sites for the TAA have demonstrated increased tumor cell binding and killing. This superior efficacy can be explained by avidity effects, whereas the monovalent CD3-binding is maintained to prevent systemic T-cell activation (2–6). For example, avidity-driven activation and killing of solid tumors was shown for the 2 + 1 bispecific TCE, AMG 509 (xaluritamig), targeting STEAP1, which allowed to discriminate between high target expressing cancer cells and normal cells (7). A first 2 + 1 TCE, glofitamab, directed against CD20 and CD3 was approved in 2023 for the treatment of patients with relapsed or refractory diffuse large B-cell lymphoma (DLBCL) (8).

Various formats are utilized to generate bispecific 2 + 1 TCEs (9, 10). Several of these formats have further been adapted for the generation of trispecific 1 + 1 + 1 TCEs targeting two different TAAs (11). From a design point of view, the generation of such 2 + 1 trispecific antibody molecules requires further engineering to allow pairing of the three different V_L_ domains with their cognate V_H_ domains. Examples of such engineering approaches include Fab-IgG molecules assembled from half-antibodies (12), Fab-IgGs comprising a common light chain (13), OrthoTsAbs built from orthogonal Fabs (14), trispecific CODV-Igs comprising a Fab moiety and a defined arrangement of V_H_ and V_L_ domains fused to C_H_1 and C_L_ domains which assemble into a bispecific binding moiety (15), and scFvs or single-domain antibodies used as building blocks (16, 17).

We have recently developed a novel technology, the eIg technology, to generate bispecific antibodies, including TCEs (18, 19). Central to this technology is the heavy chain domain 2 of the IgE (EHD2) which naturally forms disulfide-stabilized homodimers acting as a hinge-like structure in the IgE. The covalent linkage is based on two disulfide bonds at the interface of the two domains formed between two different cysteine residues. This EHD2 can be used as a versatile building block to generate homodimeric fusion proteins (20). Substitution of one of the two cysteine residues in the first EHD2 (hetEHD2-1) and substitution of the other cysteine residue in the second EHD2 (hetEHD2-2), e.g. by serine residues, results in efficient formation of disulfide-linked hetEHD2–1 x hetEHD2–2 heterodimers, while homodimers lacking disulfide bonds are instable (21). These heterodimerizing hetEHD2–1 and hetEHD2–2 domains were developed further, generating Fab-like moieties (eFab) as versatile building blocks for the generation of bispecific antibody molecules. Thus, bispecific bivalent molecules (eIg) were generated by fusing a natural Fab and an eFab moiety to heterodimerizing Fc-chains (18). Furthermore, trivalent bispecific molecules, so-called 2 + 1 formats, were generated by fusing an additional Fab to one of the eIg chains (i.e. the N- or C-terminus of one of the heavy chains or one of the light chains) (19).

In the present study, we have extended the eIg technology to generate trispecific eFab-eIg molecules comprising one natural Fab arm and two different eFab arms (1 + 1 + 1 format). The first (inner) eFab arm comprises the hetEHD2–1 domain in the heavy chain and the corresponding hetEHD2–2 domain in one of the light chains, while in the second eFab (outer) this arrangement is reversed. Recently, we have published a bivalent bispecific antibody for dual-targeting of HER2 and HER3 and confirmed strong activity against tumor cells *in vitro* and *in vivo* (22). Based on the excellent inhibitory effect of this bispecific antibody, the feasibility was evaluated for a trispecific eFab-eIgs TCE targeting HER2, HER3 and CD3. The Fc part for the generation of bi-or trispecific TCEs is silenced (FcΔab) and is not able to exert Fc-mediated effector functions (23). This trispecific eFab-eIg TCE was compared to bispecific eIgs targeting both HER2 or HER3 with respect to binding of antigen and antigen-expressing tumor cell lines and to CD3 for T-cell engagement. Finally, dual targeting of both antigens and efficient killing of HER2 and HER3 expressing tumor cells was demonstrated using 2D and 3D cell culture models.

## Materials and methods

### Materials

For the different *in vitro* experiments, we used BT474 cells (ATCC HTB-20), LIM1215 (Sigma-Aldrich Cat#10092301), MDA-MB-468 (CLS Cat#C0006003) and Jurkat cells (provided by Dr. Ammon Altman form the La Jolla Institute for Allergy & Immunology) were cultivated in RPMI 1640, 10% FCS. For the production of antibodies, we used HEK293-6E cells provided by National Research Council of Canada (Ottawa, Canada) and cultivated in F17 Freestyle expression medium (ThermoFisher) supplemented with 0.1% Kolliphor P-118 (Sigma), 4 mM GlutaMAX (ThermoFisher), and 25 μg/ml G418. Human peripheral blood mononuclear cells (PBMC) were isolated from buffy coat of healthy donors (Klinikum Stuttgart/Institut für Klinische Transfusionsmedizin und Immungenetik Ulm gemeinnützige GmbH, Germany) by Ficoll density gradient centrifugation (Lymphocyte Separation Medium 1077, Promocell, C-44010) and cultivated in RPMI 1640, 10% FCS.

### Antibody production and purification

All antibodies were produced in HEK293-6E cells cultivated in F17 Freestyle medium (ThermoFischer). Transient transfection with pSecTagA vectors carrying the genes for light and heavy chains of the different antibodies was performed with polyethyleneimine (PEI; 25 kDa, linear, Polysciences). 24 h after transfection, 2.5% (v/v) TN1 (20% (w/v) tryptone N1 (Organotechnie S.A.S.) in F17 Freestyle expression medium) was added and cells were incubated for additional four days before supernatant harvest and antibody purification via protein A affinity chromatography (Cytiva). Bound antibodies were eluted using 100 mM glycine pH 3.5 and dialyzed against phosphate-buffered saline at 4°C. A preparative FPLC size-exclusion chromatography (SEC) step was included for the eIg molecules targeting HER2xCD3 and eIg HER3xCD3.

### Antibody characterization

Purified antibodies were analyzed by SDS-PAGE (3 µg for non-reducing, 6 µg for reducing conditions) using 12% (v/v) polyacrylamide gels and staining proteins with Coomassie-Brilliant Blue G-250. Analytical SEC was performed using a VANQUISH (Thermo Fisher Scientific GmbH) HPLC in combination with a TSKgel SuperSW mAb HR column (Tosoh Bioscience) at a flow rate of 0.5 or 0.4 mL/min using 0.1 M Na_2_HPO_4_/NaH_2_PO_4_, 0.1 M Na_2_SO_4_, pH 6.7 as mobile phase. Standard proteins: thyroglobulin (669 kDa, R_S_ 8.5 nm), apoferritin (443 kDa, R_S_ 6.1 nm), β-amylase (200 kDa, R_S_ 5.4 nm), bovine serum albumin (67 kDa, R_S_ 3.55 nm) and carbonic anhydrase (29 kDa, R_S_ 2.35 nm). For eIg HER2xCD3 and eIg HER3xCD3 we further purified the molecules with a preparative SEC by FPLC. The thermal stability of molecules was analyzed by dynamic light scattering (DLS) using ZetaSizer Nano ZS (Malvern). Purified proteins were exposed to increasing temperature (30°C to 85°C) in 1°C intervals with 2-minute equilibration steps. The aggregation point was defined by the starting point of the increase in the mean count rate.

### Enzyme-linked immunosorbent assay

High-binding 96-well plates were coated with 3 µg/mL HER2-moFc and HER3-moFc (22) at 4°C overnight. Residual-binding sites were blocked with 2% (w/v) skim milk in PBS (MPBS). Antibodies were titrated (1:4) in MPBS starting from 400 nM (sequential binding of eFab-eIg) or 100 nM (binding of eFab-eIg and eIg molecules) and incubated for 1 h at RT. A human Fc-specific HRP-conjugated secondary antibody (A0170, Sigma Aldrich) was added for detection of bound antibodies or a mouse anti-His HRP-conjugated secondary antibody (9991, Cell Signaling) for detection of bound receptors and incubated for 1 h at RT. TMB was used as substrate (1 mg/mL TMB; 0.006% (v/v) H_2_O_2_ in 100 mM Na-acetate buffer, pH 6.0) and reaction was stopped using 50 µL 1 M H_2_SO_4_ and absorption was measured at a wavelength of 450 nm.

### Flow cytometry analysis

Serial dilutions of antibodies in PBA (PBS + 2% (v/v) FCS + 0.02% (w/v) sodium azide) were added to a 96-well U-bottom plate containing 1x10^5^ CD3-expressing Jurkat or HER2- and HER3-expressing tumor cells (BT474, LIM1215, MDA-MB-468) per well. Bound antibodies were detected using a R- phycoerythrin (PE)-labeled anti-human Fc antibody (109-115-098, Jackson ImmunoResearch) diluted in PBA. For the sequential binding of HER2 and HER3 using the trispecific antibody bound to Jukrat cells, we detected the receptors with an FITC-labeled anti-murine IgG antibody (F0257; Sigma Aldrich). Before every step, cells were washed three times via centrifugation at 500 x g/4°C for 3 min and resuspension in 150 µL PBA. Binding of the antibodies to the cells was analyzed using a MACSQuant VYB (Miltenyi Biotec) and FlowJo (BD Biosciences). The relative median fluorescence intensity (MFI) was calculated as followed: relative MFI = ((MFI_sample_-(MFI_detection_-MFI_cells_))/MFI_cells_).

### 2D cytotoxicity assay

Tumor cells (7,500 to 15,000 cells/well) were seeded per 96-well in RPMI containing 10% (v/v) FCS and P/S (1:100) and were incubated 24 h at 37°C/5% (v/v) CO_2_. In addition, PBMCs were thawed and cultivated in a cell culture dish (10 cm) in 10 mL RPMI with 10% (v/v) FCS overnight. Serial dilutions of tri- or bispecific antibodies in RPMI containing 10% (v/v) FCS and P/S (1:100) were pre-incubated with the tumor cells for 15 min. Subsequently, PBMCs from different donors were added to the tumor cells in an effector-to-target ratio of 10:1 and incubated for 3 days. Supernatant was then discarded and remaining viable tumor cells were stained with crystal violet and optical density at 550 nm was measured using the Tecan Spark (Tecan).

### 3D spheroid killing assay

BT474 cells (1,000 cells/well) were seeded on poly-HEMA coated U-bottom 96-well plates to prevent cell attachment in RPMI + 10% (v/v) FCS + P/S (1:100) and left 24 h at 37°C/5% (v/v) CO_2_ to form compact spheroids. In addition, PBMCs were thawed and cultivated in a cell culture dish (10 cm) in 10 mL RPMI with 10% (v/v) FCS overnight. The next day, spheroids were treated with different dilutions of tri- or bispecific antibodies as well as 1 µg/mL PI and pre-incubated for 15 min at 37°C/5% (v/v) CO_2_. Different numbers of PBMCs were added to the spheroids. Target cell killing was observed via PI staining intensity at the IncuCyte every 2 h for two days. Images were analyzed using Fiji (Open Source).

### IL-2, IFNγ, IL-6 and TNFα release

Target cells were incubated with bi- or trispecific antibodies and PBMCs at an effector-to-target ratio of 10:1. After 24 h or 48 h, supernatants were harvested for quantification of IL-2, IL-6 and TNFα or IFNγ, respectively. Supernatant cytokine levels were quantified via by sandwich ELISA following the manufacturer’s instructions (IL-2/IFNγ/TNFα Duo Set ELISA; R&D Systems; ELISA MAX Standard Set human IL-6; BioLegend).

### Statistics

All data are represented as mean ± SD for n=3 if not indicated otherwise. For co-culture experiments analyzing the T-cell activation, two different donors were tested. Significances were analyzed with GraphPad Prism 8 using an unpaired two-tailed t test for the analysis of two samples or a one-way ANOVA followed by Tukey multiple comparison test (posttest) for the analysis of more than two samples.

## Results

### Generation of bivalent bispecific and trivalent trispecific antibodies

**Figure 1:**
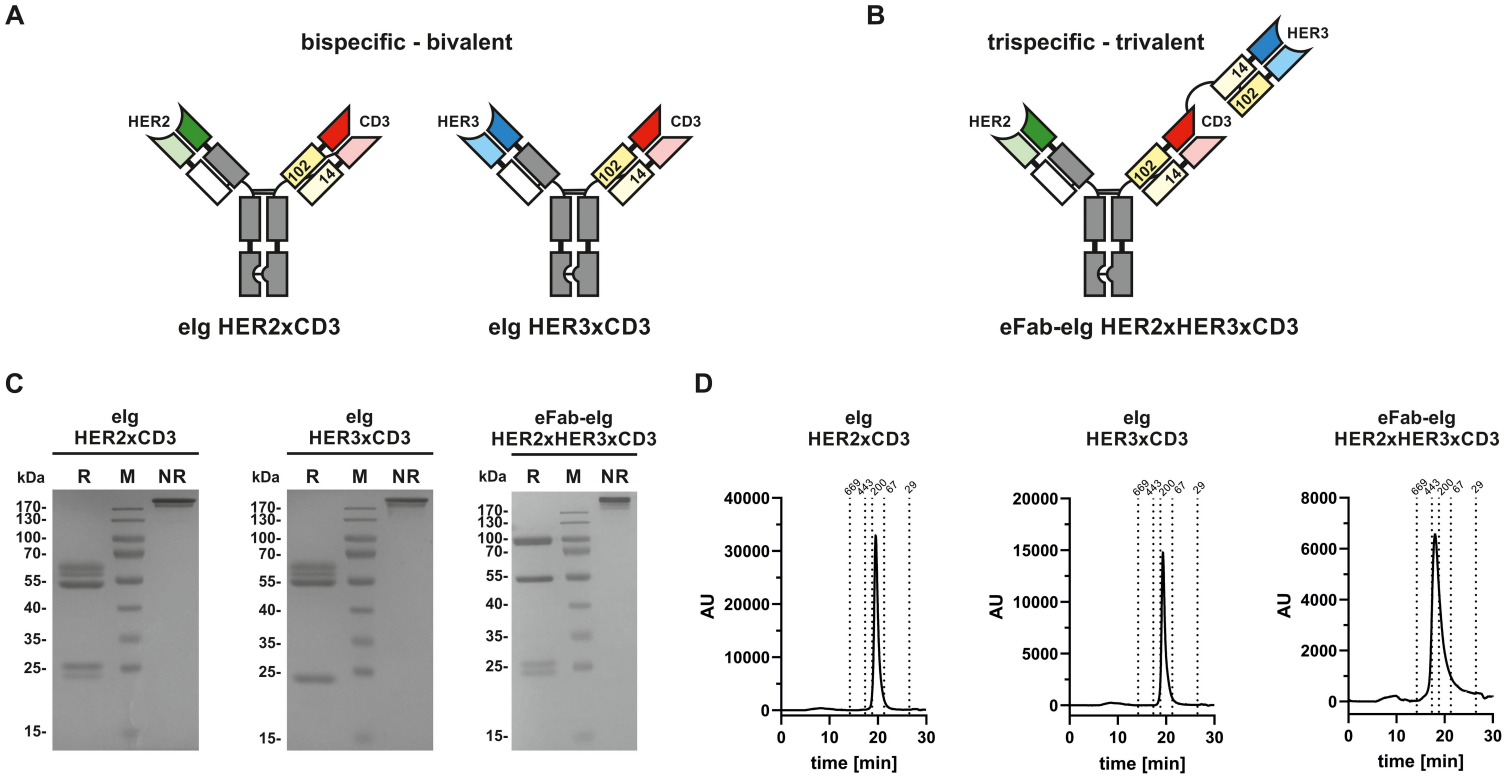
Composition and biochemical analysis of bi- and trispecific molecules. **(A)** Composition of bispecific bivalent eIg molecules directed against HER2 and CD3 as well as HER3 and CD3, respectively. **(B)** Composition of trispecific trivalent eFab-eIg molecules directed against HER2, HER3 and CD3. **(C)** SDS-PAGE of bispecific bivalent eIg molecules and trispecific trivalent molecule under reducing (R) and non-reducing (NR) conditions. **(D)** SEC analysis of eIg HER2xCD3 and HER3xCD3 as well as of eFab-eIg HER2xHER3xCD3.

A trispecific, trivalent eFab-eIg molecule was generated by fusing a Fab directed against HER2 [derived from trastuzumab (24)] to a Fc-hole chain and a tandem arrangement of eFabs (eFab1-eFab2) to a Fc-knob chain. The first (inner) eFab-1 is directed against CD3 using a humanized version of UCHT1 (25) and the second (outer) eFab-2 derived from the antibody 3–43 is directed against HER3 (26). In this design, each eFab comprises two heterodimerizing EHD2 (hetEHD2 domains substituting C_H_1 and C_L_). In the eFab1, the heavy chain hetEHD2 carries a C14S mutation, thus possessing only C102 at the interphase, while the light hetEHD2 carries a C102S mutation, thus possessing only C14 at the interphase. In eFab2 these mutations are reversed. As control proteins, bispecific, bivalent eIg molecules directed against HER2 and CD3, or HER3 and CD3, respectively, were generated (**Figures 1A, B**). All three molecules were produced in transiently transfected HEK293-6E cells and purified from the supernatant with yields of 6.4 mg/L supernatant for the eFab-eIg, 6.7 mg/L for eIg HER2xCD3, and 9.2 mg/L for eIg HER3xCD3. SDS-PAGE analyses confirmed correct assembly into eIg molecules and the presence of the individual polypeptide chains (**Figure 1C**). Purity of >95% was further confirmed by size-exclusion chromatography showing a single main peak with an apparent molecular mass of approximately 190 to 200 kDa for both eIgs and approximately 310 kDa for the eFab-eIg (**Figure 1D**). In addition, we have also tested the thermal stability of the different molecules by dynamic light scattering (DLS) (**Supplementary Figure S1**). Here, we calculated an aggregation point of the trispecific eFab-eIg molecule with 75°C (as well as for trastuzumab, IgG huU3 and eIg HER2xCD3), while one of the parental monospecific antibody IgG 3–43 and the bispecific eIg HER3xCD3 showed a lower aggregation point at 64°C and 63°C, respectively. Thus, the aggregation point originating from variable domains of targeting HER3 was not observed for the trispecific eFab-eIg molecule.

### Binding of bi- and trispecific antibodies to HER2 and HER3

**Figure 2:**
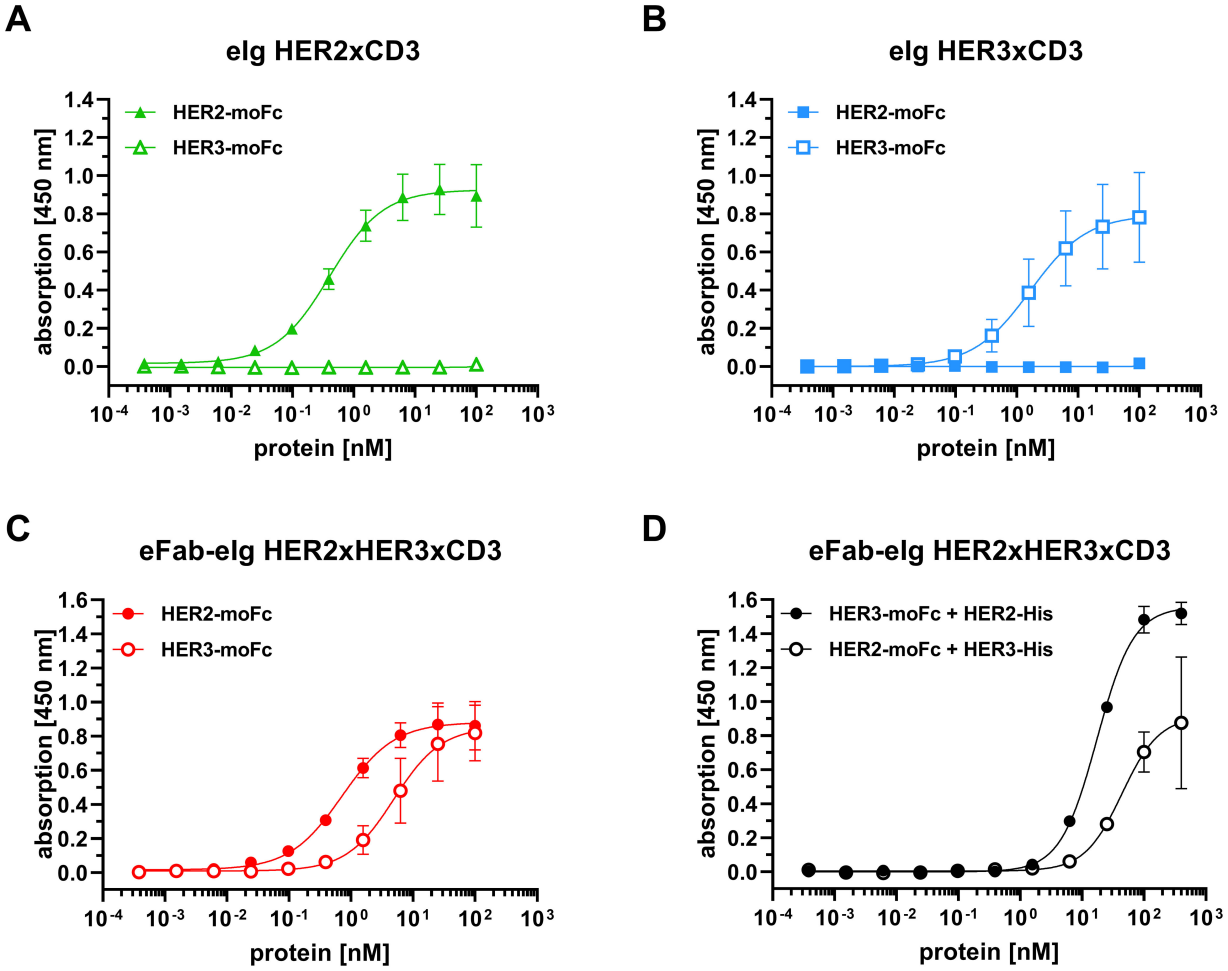
Binding of bi- and trispecific molecules to HER2 and HER3 in ELISA. **(A)** Binding of eIg HER2xCD3 to HER2 and HER3. **(B)** Binding of eIg HER3xCD3 to HER2 and HER3. **(C)** Binding of eFab-eIg HER2xHER3xCD3 to HER2 and HER3. **(D)** Binding of HER3-His to eFab-eIg HER2xHER3xCD3 bound to immobilized HER2-moFc, or vice versa. Mean ± SD; n=3.

First, binding of the bi- and trispecific eIg antibodies was analyzed by ELISA using immobilized recombinant extracellular regions of HER2 and HER3 fused to a mouse Fc region (HER2-moFc and HER3-moFc). In this assay, all antibodies showed a concentration-dependent binding (**Figures 2A-C**) with EC_50_ values of 0.7 nM for HER2 and 5.6 nM for HER3 for the trispecific eFab-eIg, 0.4 nM for HER2 for eIg HER2xCD3 and 1.9 nM for HER3 for eIg HER3xCD3. Thus, compared to the bispecific eIgs, the EC_50_ values of the trispecific eFab-eIg were slightly lower than those observed for the bispecific antibodies (**Table 1**). A significantly different binding was calculated for the trispecific eFab-eIg molecule compared to the bispecific eIg HER2xCD3 molecule (p=0.03). Furthermore, binding to both antigens was analyzed by a sandwich ELISA using either immobilized HER2-moFc or HER3-moFc followed by incubation with the eFab-eIg HER2xHER3xCD3 and subsequent incubation with either HER3-His or HER2-His, respectively (**Figure 2D**). In both setups, binding to both antigens was observed for the trispecific eFab-eIg.

**Table 1:** Antigen binding from ELISA experiments.

Antigen	eIg HER2xCD3	eIg HER3xCD3	eFab-eIg HER2xHER3xCD3
HER2-moFc	0.4 ± 0.1	n.d.	0.7 ± 0.2
HER3-moFc	n.d.	1.9 ± 1.1	5.6 ± 2.1

EC_50_ values in nM. Mean ± SD, n.d., not determined, n=3.

### Binding of bi- and trispecific antibodies to CD3

**Figure 3:**
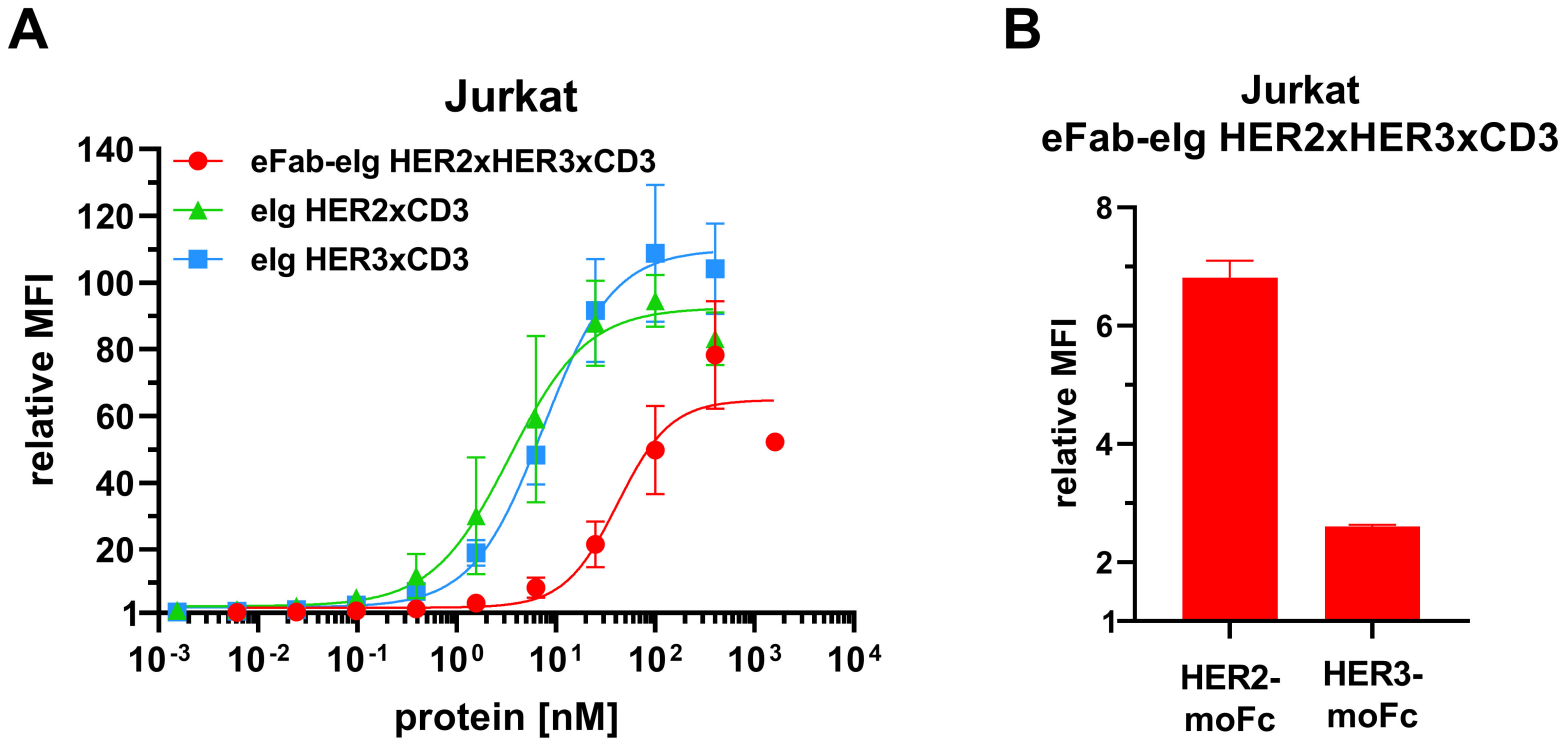
Binding to CD3-expressing Jurkat cells. **(A)** Flow cytometry analysis of binding of eFab-eIg HER2xHER3xCD3 as well as eIg HER2xCD3 and HER3xCD3 to CD3-expressing Jurkat cells. **(B)** Flow cytometry analysis of binding of 400 nM of eFab-eIg HER2xHER3xCD3 to Jurkat cells followed by incubation with either HER2-moFc or HER3-moFc (300 nM), respectively, to analyze sequential binding to CD3-expressing cells and either HER2 or HER3. Mean ± SD, n=3.

Next, antibody binding to CD3 was analyzed by flow cytometry using CD3-expressing Jurkat cells. For all antibodies a concentration-dependent binding was observed (**Figure 3A**). The trispecific eFab-eIg HER2xHER3xCD3 bound to the cells with an EC_50_ value of 45.8 nM, while the control antibodies bound with an EC_50_ value of 7.0 nM for eIg HER2xCD3 and 4.4 nM for eIg HER3xCD3 (**Table 2**). The reduced binding of eFab-eIg HER2xHER3xCD3, which reached significance (p<0.004 for eIg HER2xCD3 and p<0.003 for eIg HER3xCD3) is most likely due to the N-terminal fusion of the eFab moieties, sterically interfering with the binding to CD3. We additionally analyzed the sequential binding of HER2-moFc or HER3-moFc to the trispecific eFab-eIg bound to Jurkat cells (**Figure 3B**). After incubation of Jurkat cells with 400 nM of eFab-eIg HER2xHER3xCD3, soluble HER2-moFc or HER3-moFc was added and bound antigens were detected with a FITC-labeled anti-murine Fc antibody. Both antigens, HER2 and HER3, were bound to the cells incubated with eFab-eIg HER2xHER3xCD3, demonstrating the sequential binding of CD3-expressing cells with HER2 or HER3 as antigen.

**Table 2:** Cell binding from flow cytometry analysis.

Cell line	eIg HER2xCD3	eIg HER3xCD3	eFab-eIg HER2xHER3xCD3
Jurkat	7.0 ± 0.6	4.4 ± 2.6	45.8 ± 14.8
MDA-MB468	n.d.	0.3 ± 0.1	7.0 ± 0.8
LIM1215	2.6 ± 0.8	0.6 ± 0.1	1.4 ± 0.4
BT474	6.2 ± 1.5	0.5 ± 0.5	10.9 ± 2.8^*^

EC_50_ values in nM. Mean ± SD, n.d., not determined, ^*^n=2, n=3.).

### Target cell binding with varying surface expression of HER2

**Figure 4:**
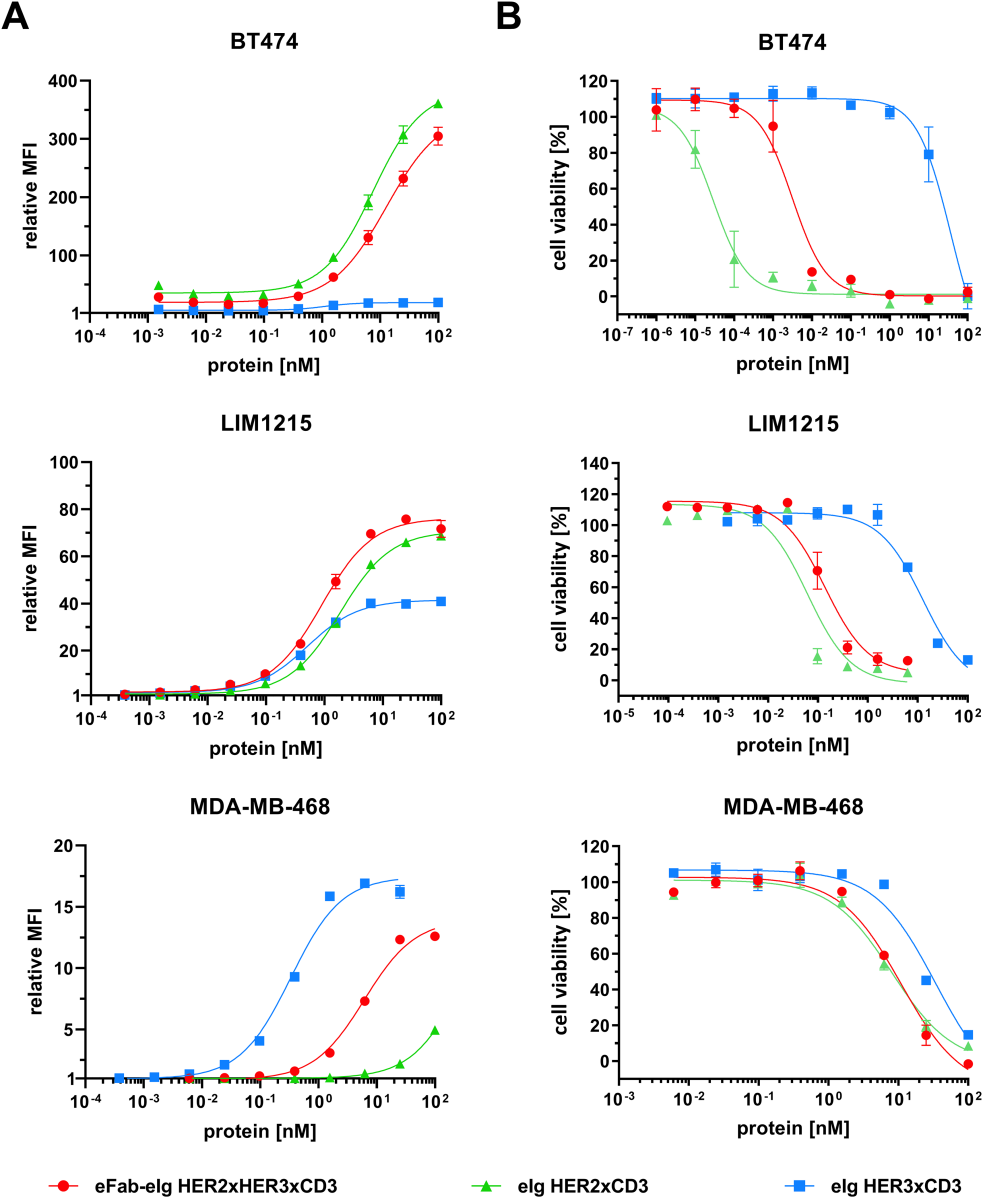
Target cell binding and cytotoxicity. **(A)** Binding of different eIg molecules (eFab-eIg HER2xHER3xCD3; eIg HER2xCD3; eIg HER3xCD3) to BT474, LIM1215 and MDA-MB-468 cells was analyzed via flow cytometry. **(B)** Killing of target cells (BT474, LIM1215 and MDA-MB-468) incubated with bi- or trispecific eIg molecules and PBMCs (donor: HN#7 for BT474; HN#6 for LIM1215 and AH#1 for MDA-MB-468) at an effector-to-target (E:T) ratio of 10:1 for 3 days. Mean of duplicates ± SD, n=1.

To investigate target cell specificity, binding of the antibodies to HER2- and HER3-expressing cell lines was analyzed by flow cytometry using tumor cell lines expressing different surface levels of HER2 and HER3 (BT474: >572,000 HER2/cell and ~11,000 HER3/cell; LIM1215: ~33,000 HER2/cell and ~20,000 HER3/cell; MDA-MB-468: ~1,700 HER2/cell and ~6,000 HER3/cell). In all cases, binding to the target cells occurred in a concentration-dependent manner (**Figure 4A**, **Supplementary Figure S2**). For all three cell lines strongest binding was observed for eIg HER3xCD3 with EC_50_ values in the range of 0.3 to 0.6 nM. For BT474 cells, which express very high amount of HER2 and comparable low amount of HER3, binding of eIg HER3xCD3 was detected with lower fluorescence intensity compared to the trispecific antibody and eIg HER2xCD3 binding to HER3 and/or HER2 and is highlighted in **Supplementary Figure S3**. The eIg HER2xCD3 bound best to LIM1215 (2.6 nM) followed by BT474 (6.2 nM) and MDA-MB-468 cells (EC_50_ value was not determined). The trispecific antibody showed similarly strong binding to LIM1215 (1.4 nM) followed by MDA-MB-468 (7.0 nM) and BT474 cells (10.9 nM). A significant difference was determined for the eIg HER3xCD3 compared to eIg HER2xCD3 using LIM1215 cells (p=0.008) and BT474 cells (p=0.016) and to trispecific antibody using BT474 cells (p=0.002) and MDA-MB-468 cells (p<0.001). In general, MFI signal intensity and thus binding efficacy correlated with HER2 and HER3 expression levels. Of note, the trispecific eFab-eIg consistently gave rise to strong signals, while maximal MFI signals of the bispecific eFabs varied, depending on the antigen expression levels. For example, eIg HER2xCD3 showed very low binding to MDA-MB-468 cells, which express low levels of HER2, while eIg HER3xCD3 showed low binding to BT474 cells. In summary, binding to all three different cancer cell lines with varying amounts of HER2 and HER3 was detected for the trispecific antibody.

### Functional anti-tumor activity of the bi- and trispecific antibodies in 2D and 3D model system

**Figure 5:**
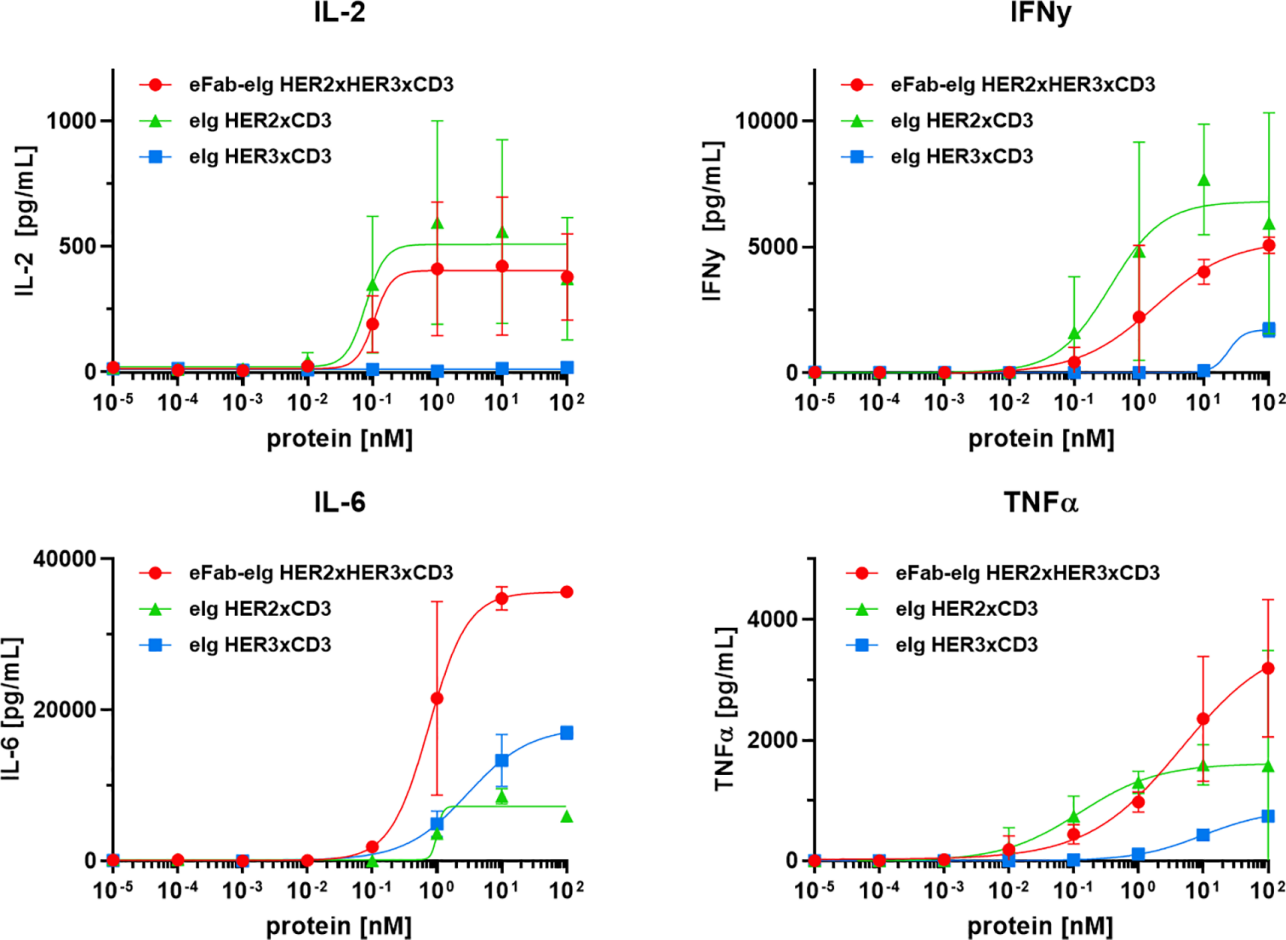
T-cell activation by TCEs. Release of IL-2, IL-6 and TNFα after 24 h and IFNγ after 48 h by PBMCs co-cultured with LIM1215 using an effector-to-target ratio of 10:1 analyzed by sandwich ELISA. Mean ± SD, n=2 - two individual donors).

To assess the cytotoxic activity of the antibodies, we co-cultured cancer cells with PBMCs at a effector to target cell ratio of 10:1 in the presence of the bi- and trispecific antibodies. (**Figure 4B**, **Supplementary Figure S4**). The bispecific eIg HER3xCD3 molecule showed the lowest activity on all tumor cell lines with EC_50_ values in the range of 12.7 to 38.8 nM, as the surface expression of HER3 of all tumor cell lines is in a similar range (from 6,000 to 20,000 HER3 receptors/cell). The HER2 expression of the different tumor cell lines strongly differs (from less than 1,700 to more than 578,000 HER2 receptors/cell) and showed strong cytotoxic effect of the bispecific HER2xCD3 and the trispecific eFab-eIg molecule with dependency of target cell binding. For MDA-MB-468 cells with low HER2 levels, EC_50_ values of 12.3 nM for the bispecific and 23.7 nM for the trispecific molecule were calculated, while stronger activity was detected for LIM1215 cells with moderate HER2 levels (EC_50_ values of 100 pM for eIg HER2xCD3 and 300 pM for eFab-eIg). Strongest killing with EC_50_ values of 0.1 pM for eIg HER2xCD3 and 2 pM for eFab-eIg were observed for the BT474 cells with high HER2 levels (**Table 3**). For BT474 cells, a significance between eIg HER3xCD3 (p=0.03) compared to the trispecific eFab-eIg and the HER2xCD3 eIg molecule was calculated. In addition, we used the T-cell activation system by using LIM1215 cells upon T-cell engagement to ensure and quantified immunostimulatory factors, like IL-2, IFNγ, IL-6 and TNFα, to analyze effects on the immune system (**Figure 5**, **Table 4**). In line with the cytotoxic activity of the bi- and trispecific antibodies, strong concentration-dependent release of IL-2, IFNγ, IL-6 and TNFα was observed for the bispecific eIg targeting HER2 and CD3, and the trispecific antibody, while the bispecific HER3xCD3 eIg triggered only a marginal cytokine response.

**Table 3:** Tumor cell killing from cell viability assay.

Cell line	eIg HER2xCD3	eIg HER3xCD3	eFab-eIg HER2xHER3xCD3
MDA-MB468	8.6 ± 2.3	14.3 ± 17.3	23.7 ± 15.0
LIM1215	0.1 ± 0.1	12.7 ± 0.1	0.3 ± 0.3
BT474	0.0001 ± 0.0001	38.8 ± 22.7	0.0022 ± 0.0017

EC_50_ values in nM. Mean ± SD, n=3.

**Table 4:** T-cell activation.

Cell line	eIg HER2xCD3	eIg HER3xCD3	eFab-eIg HER2xHER3xCD3
IL-2	0.08 ± 0.03	n.d.	0.1 ± 0.01
IFN-γ	0.7 ± 0.7	50.3 ± 49.9	3.4 ± 4.4
IL-6	1.0 ± 0.01	5.1 ± 5.1	1.0 ± 0.8
TNFα^*^	0.1	9.7	4.6

EC_50_ values in EC_50_ values in nM. Mean ± SD, n.d., not determined, n=2; ^*^calculation of EC_50_ was based on both experiments.

**Figure 6:**
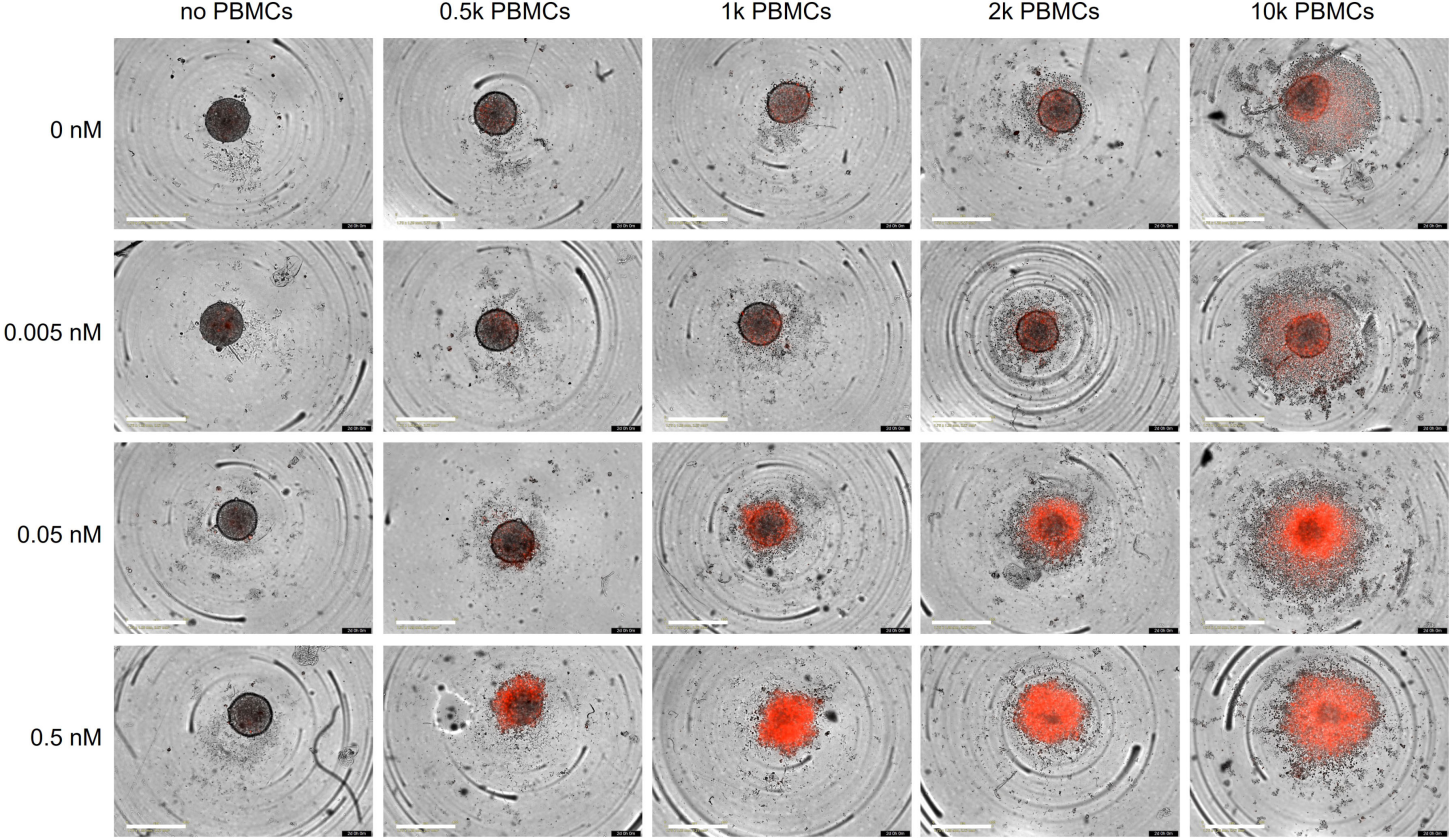
Killing of BT474 spheroids. BT474 spheroids with a diameter of approximately 250 µm were used as target cells and incubated with different amount of trispecific eFab-eIg antibodies as well as different number of PBMCs (donor: AL#1). In addition, the induction of apoptosis was analyzed using propidium iodide (PI; 1 µg/mL). Cells were incubated with antibodies and the PBMCs for 48 h at 37°C. n=1.

Finally, we employed a 3D co-culture assay to evaluate the cytotoxic activity of the trispecific antibody under conditions that more closely mimic the situation found *in vivo*. We first generated cancer cell spheroids by seeding BT474 cells into ultra-low attachment plates. Once they reached a size of approximately 250 µm, spheroids were incubated with varying numbers of PBMCs/well and different concentrations of the trispecific eFab-eIg for up to 2 days (**Figure 6**). Dead cells were visualized by PI staining. In the absence of PBMCs, the antibody neither had effects on spheroid morphology, nor did it induce cell killing. In agreement with the 2D experiments, cell death was effectively induced in the presence of PBMCs, to a greater extent and with faster kinetics when using higher numbers of PBMCs and higher antibody concentrations. In summary, our data demonstrate that the trispecific antibody showed strong T-cell activation and subsequential tumor cell killing in the 2D and 3D model system.

## Discussion

Here, we have advanced the eIg technology to generate trispecific trivalent antibodies with an extended Ig-like structure. The underlying design principle utilizes heterodimerizing EHD2 domains derived from the homodimerizing heavy chain domain 2 of IgE (EHD2) to generate Fab-like building blocks (eFabs). The EHD2 homodimers are normally covalently linked by two disulfide bonds formed between C14 and C102. Substituting Cys14 in a first EHD2 (EHD2-1) and C102 in a second EHD2 (EHD2-2) by serine residues results in efficient heterodimerization since only heterodimers are capable of forming a single disulfide bond while homodimers cannot do so and are thus unstable (18, 20). These hetEHD2 domains are used to replace C_H_1 and C_L_ in a normal Fab to obtain Fab-like moieties (eFabs). In comparison to other antibody fragments, e.g. scFv molecules, as building blocks for multispecific antibodies, the usage of the disulfide-linked constant domains in the eIg technology increases the thermal stability and the solubility of multispecific molecule and lowers the potential for aggregations (27, 28). In the present study it was found that placing the EHD2–1 and EHD2–2 domains on different chains mediates correct pairing of cognate V_H_ and V_L_ in co-expressed eFab-1 and eFab-2 moieties without further modifications of the variable domains. Proof of concept was obtained for a trispecific eFab-eIg targeting HER2, HER3 and CD3. The molecule retained binding activity for its target antigens and was capable of recruiting T-cells to tumor cells and mediating T-cell-induced killing of tumor cell lines with varying levels of HER2 and HER3 expression.

The majority of trispecific antibodies in preclinical and clinical development aim at directing immune effector cells to tumor cells (10). Combining T-cell engagement with the targeting of two different surface antigens can result in improved tumor selectivity by avidity-driven on-target activity. This was, for example, shown for a trispecific TCE targeting Lys6E, B7-H4 and CD3, mediating strong killing of breast cancer cells simultaneously expression Ly6E and B7-H4 *in vitro* and *in vivo* (12). Furthermore, a trispecific TCE targeting EGFR and a NY-ESO-1 showed strongly increased efficacy and anti-tumor activity compared to TCEs targeting single antigens (29). In another study, Tapia-Galisteo and coworkers used a trispecific (EGFRxEpCAMxCD3) TCE and modified the affinity for both TAAs. For EGFR- and EpCAM-positive HCT116 cells, a 100-fold increased cell killing activity was detected compared to EGFR-positive or EpCAM-positive tumor cells (30). However, the trispecific eFab-eIg molecule described here did not exhibit increased cell binding and cytotoxicity compared to the 1 + 1 bispecific TCEs targeting either HER2 or HER3, but rather combined both activities within one molecule without causing an avidity-driven increase of activity. This can be explained by sterical hindrance, impairing the simultaneous binding to HER2 and HER3 on the same cell, and further interacting with CD3 on T-cells. Indeed, we previously reported that the format matters, i.e. the geometry and architecture of trivalent bispecific TCEs targeting EGFR affected the degree of target cell killing by the T-cells (19). Since CD3 binding by the inner eFab was hampered by the outer eFab targeting HER3, as shown by flow cytometry assays using Jurkat cells, in future studies trispecific molecules with altered arrangements of the three binding sites might identify formats with increased binding and activity.

Nevertheless, the trispecific eFab-eIg TCE molecule was capable of efficiently killing tumor cells with varying levels of HER2 and HER3. Specifically, BT474 cells which express high levels of HER2 and low levels of HER3, and therefore are less susceptible to HER3 targeting alone, were efficiently killed by the trispecific TCE. HER3 expression is often elevated as a compensation mechanism in HER2-resistant tumor cells (31, 32). It is known that the HER family of receptor tyrosine kinases displays a high degree of plasticity which can provide compensatory signaling associated with acquired resistance to treatment (33). Thus, HER3 can be expressed as a compensatory signal in HER2-resistant tumor cell lines. In such settings, a trispecific TCE that targets both HER2 and HER3 might prove beneficial, by preventing or prolonging the development of acquired resistance.

The beneficial effects of dual targeting are further supported by findings for a DNA-encoded trispecific TCE targeting IL13Rα2, EGFRvIII and CD3. This trispecific TCE was designed to address the tumor heterogeneity of glioblastoma and demonstrated efficient tumor growth control in a GBM model with heterogenous expression of IL13Rα2 and EGFRvIII, resembling the complex tumor environment in human GBM (34). In addition, the superiority of dual targeting TCEs was demonstrated for a trispecific TCE targeting CD3, BCMA, and CD38 (ISB 2001), utilizing a common-light chain approach to generate a trispecific Fab-IgG molecule (35).

Furthermore, trispecific antibodies allow to address tumor escape mechanisms, e.g. resulting from the downregulation or loss of a target antigen. For example, treatment with the bispecific TCE blinatumomab targeting CD19 and CD3 resulted in the appearance of CD19-negative leukemic blasts in approximately 30% of patients (36). This antigen loss was circumvented by integration of binding sites for a CD20 fragment (37) or CD22 (38) as a second tumor cell targeting element. Similarly, down-regulation of HER2 was detected in cell lines treated with a trastuzumab-ADC T-DM1 as well as in four patients after receiving the dual trastuzumab/pertuzumab combination therapy (39). Adding a HER3 binding site to a HER2-targeting TCE could address the tumor heterogeneity and exploit the potential emergence of HER3 expression as compensatory signal (33, 40). Of note, a first bispecific antibody (zenocutuzumab) targeting HER2 and HER3 was recently approved for cancer therapy (41).

In summary, by adapting our eIg technology, we were capable of generating a trivalent trispecific eFab-eIg molecule for T-cell engagement. Using two different eFab building blocks (eFab-1, eFab-2) together with a normal Fab moiety allows the generation of trispecific molecules with varying valency and geometry. This was also exemplarily shown for bispecific eIg variants targeting EGFR and CD3, either comprising or lacking an Fc region (19). Thus, the eIg technology represents a versatile platform for the generation multispecific antibodies for a multitude of applications.

## Data Availability

The raw data supporting the conclusions of this article will be made available by the authors, without undue reservation.

## Supplementary material

Supplementary Figure 1 Thermal stability. Determination of the aggregation point by dynamic light scattering (DLS) of the purified Fab-eIg, eIg and IgG molecules. Dotted line indicates the aggregation point. n=1.

Supplementary Figure 2 Target cell binding. Binding of eIg molecules (eFab-eIg HER2xHER3xCD3; eIg HER2xCD3; eIg HER3xCD3) to BT474, LIM1215 and MDA-MB-468 cells was analyzed via flow cytometry. Mean ± SD, N=2 and 3.

Supplementary Figure 3 Zoom in for binding of eIg HER3xCD3 to BT474 cells. Binding analysis of eIg HER3xCD3 to BT474 from **Figure 4** and **Supplementary Figure S1** was used to generate a zoom in.

Supplementary Figure 4 Target cell cytotoxicity. Killing of target cells (BT474, LIM1215 and MDA-MB-468) incubated with bi- or trispecific eIg molecules and PBMCs (AL#1 and AH#2 for BT474; AH#1 and HN#4 for LIM1215 as well as HN#4 and HN#6 for MDA-MB-468) at an effector-to-target (E:T) ratio of 10:1 for 3 days. Mean ± SD, N=2 and 3.
