# Supplementary figures and images for: Trispecific eFab-eIg T-cell engagers targeting HER2 and HER3
Source: Front Immunol. 2025 Aug 27;16:1642454. doi: 10.3389/fimmu.2025.1642454 (PMC12420622; doi:10.3389/fimmu.2025.1642454)

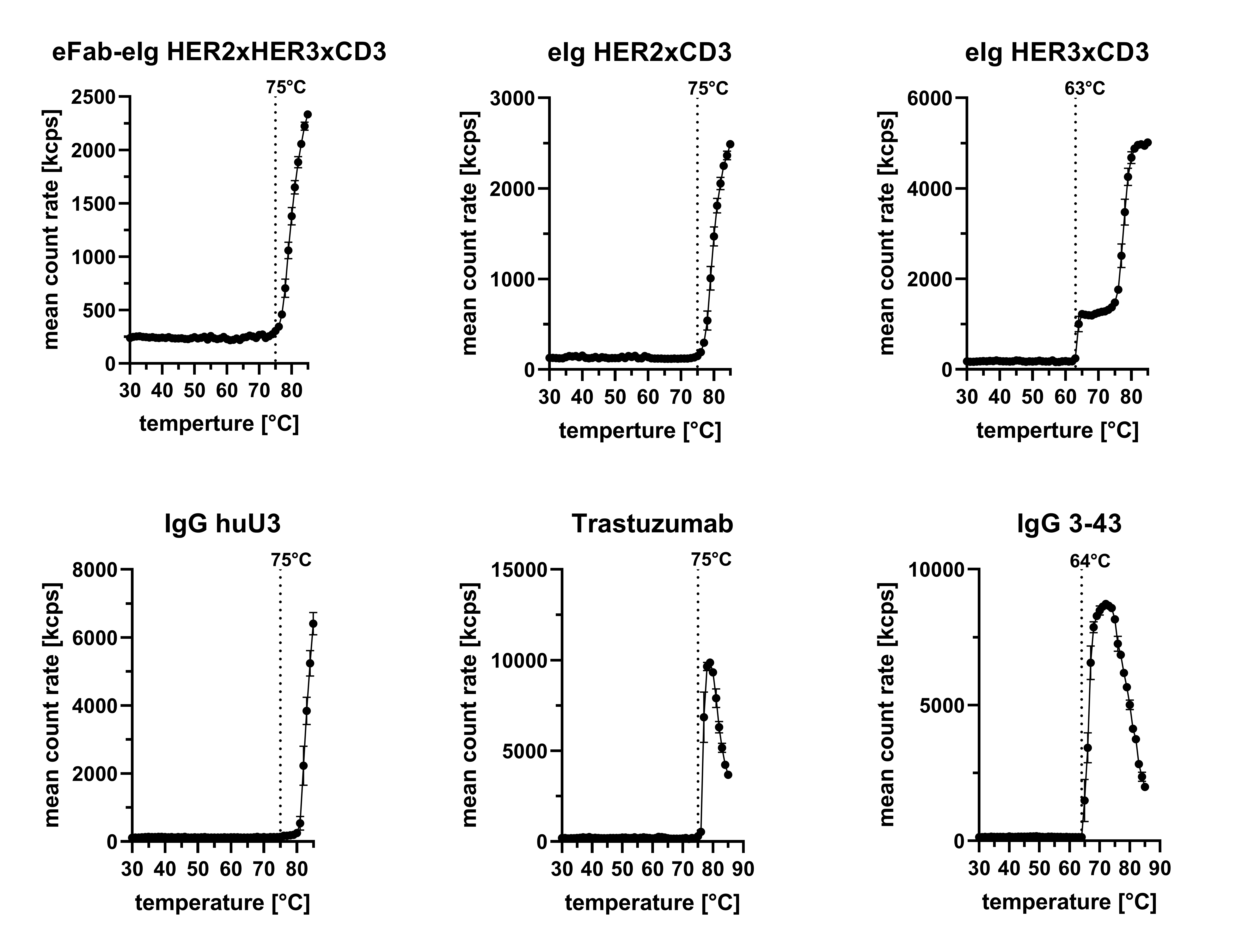

Supplement: Supplementary Figure 1 — Thermal stability. Determination of the aggregation point by dynamic light scattering (DLS) of the purified Fab-eIg, eIg and IgG molecules. Dotted line indicates the aggregation point. n=1. [file DataSheet1.zip › Figure S1.tif]

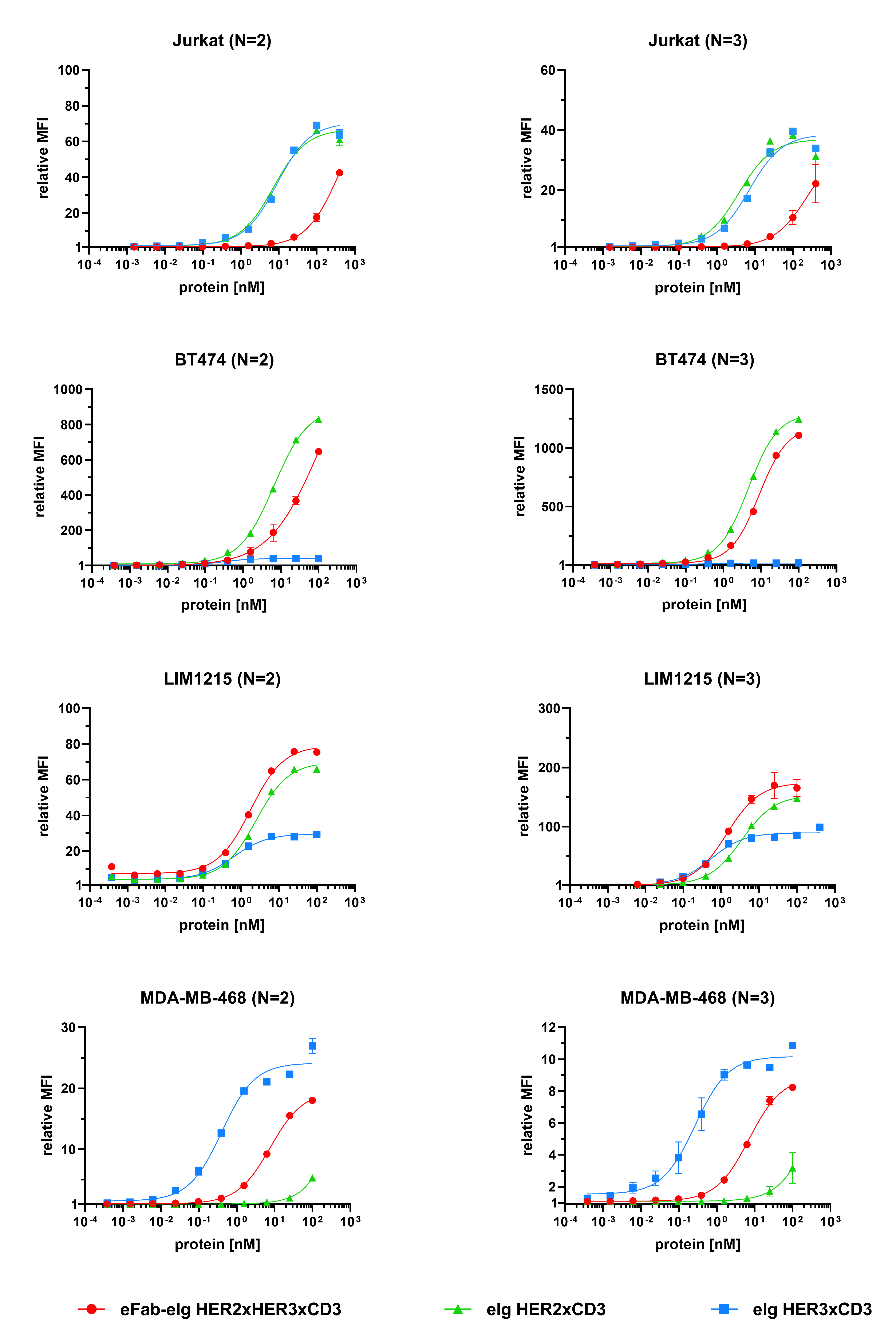

Supplement: Supplementary Figure 1 — Thermal stability. Determination of the aggregation point by dynamic light scattering (DLS) of the purified Fab-eIg, eIg and IgG molecules. Dotted line indicates the aggregation point. n=1. [file DataSheet1.zip › Figure S2.tif]

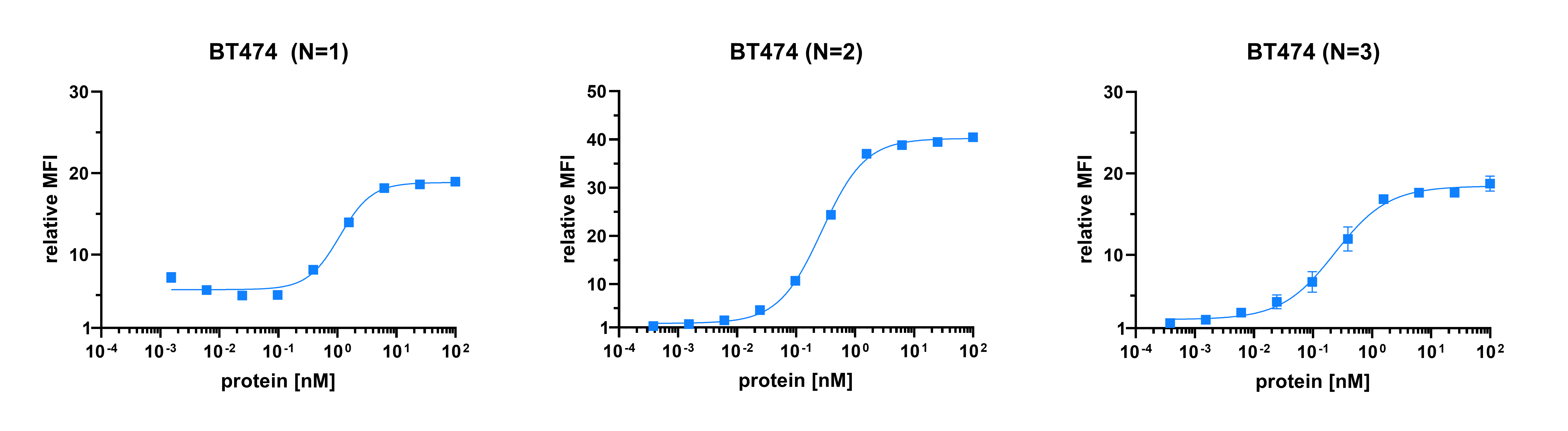

Supplement: Supplementary Figure 1 — Thermal stability. Determination of the aggregation point by dynamic light scattering (DLS) of the purified Fab-eIg, eIg and IgG molecules. Dotted line indicates the aggregation point. n=1. [file DataSheet1.zip › Figure S3.tif]

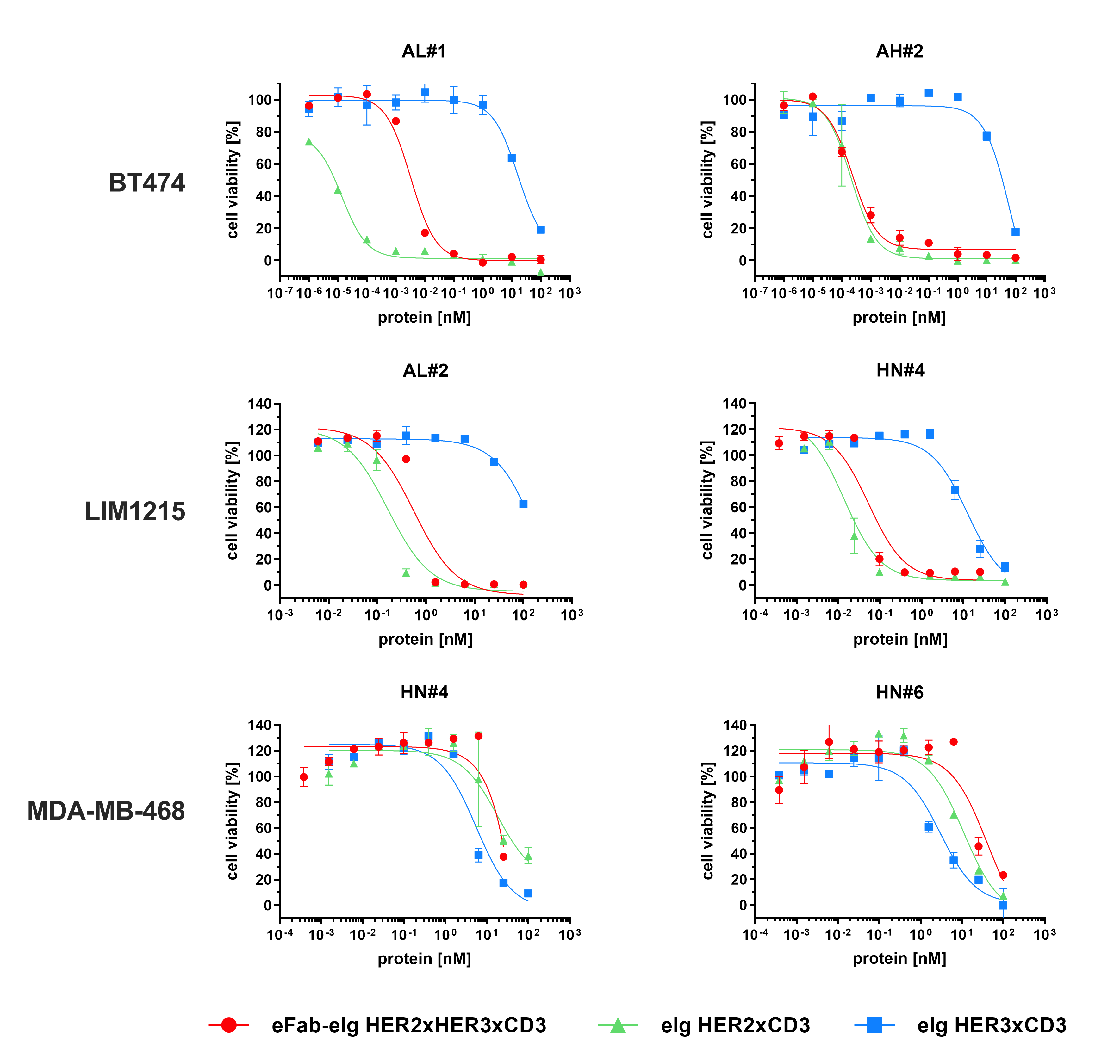

Supplement: Supplementary Figure 1 — Thermal stability. Determination of the aggregation point by dynamic light scattering (DLS) of the purified Fab-eIg, eIg and IgG molecules. Dotted line indicates the aggregation point. n=1. [file DataSheet1.zip › Figure S4.tif]
